# Effect of the Heat-Treated Ti6Al4V Alloy on the Fibroblastic Cell Response

**DOI:** 10.3390/ma11010021

**Published:** 2017-12-30

**Authors:** Mercedes Paulina Chávez-Díaz, María Lorenza Escudero-Rincón, Elsa Miriam Arce-Estrada, Román Cabrera-Sierra

**Affiliations:** 1 Instituto Politécnico Nacional, Departamento de Ingeniería en Metalurgia y Materiales, UPALM Edificio 7, Mexico City 07738, Mexico; mpaulinachavezdiaz@yahoo.com.mx (M.P.C.-D.); earce@ipn.mx (E.M.A.-E.); 2 Centro Nacional de Investigaciones Metalúrgicas (CENIM-CSIC), Departamento de Ingeniería de Superficies, Corrosión y Durabilidad, Madrid 28040, Spain; escudero@cenim.csic.es; 3 Instituto Politécnico Nacional, Departamento de Ingeniería Química Industrial, UPALM Edificio 7, Mexico City 07738, Mexico

**Keywords:** Ti6Al4V, heat treatment, fibroblasts

## Abstract

Two heat treatments were carried out below (Ti6Al4V_800_) and above (Ti6Al4V_1050_) Ti6Al4V beta-phase transformation temperature (980 °C), with the purpose of studying the effect of microstructure on the adhesion and proliferation of fibroblast cells, as well as their electrochemical behavior. These alloys were seeded with 10,000 L929 fibroblast cells and immersed for 7 days in the cell culture at 37 °C, pH 7.40, 5% CO_2_ and 100% relative humidity. Cell adhesion was characterized by Scanning Electron Microscopy (SEM) and Electrochemical Impedance Spectroscopy (EIS) techniques. Polygonal and elongated cell morphology was observed independent of Ti6Al4V microstructure. Besides, C, O, P, S, Na and Cl signals were detected by Energy Dispersive X-Ray Spectroscopy (EDX), associated with the synthesis of organic compounds excreted by the cells, including protein adsorption from the medium. In certain areas on Ti6Al4V and Ti6Al4V_800_ alloys, cells were agglomerated (island type), likely related to the globular microstructure; meanwhile, larger cellular coverage is shown for Ti6Al4V_1050_ alloy, forming more than one layer on the surface, where only Ca was recorded. Impedance diagrams showed a similar passive behavior for the different Ti6Al4V alloys, mainly due to TiO_2_ overlaying the contribution of the organic compounds excreted by fibroblast cells.

## 1. Introduction

Commercially pure titanium (Ti-CP) and alpha-beta alloys, such as Ti6Al4V, are widely used as structural materials for replacement of hard tissues, hip joints and dental implants due to their excellent mechanical strength, corrosion resistance, and biocompatibility [1]. Biocompatibility and the success of endoprosthetic devices depend on biological processes, such as cell adhesion, proliferation and differentiation, occurring on the metal surface prior to osseointegration [2]. These cellular processes are affected by the surface hydrophilicity, the arrival of ions at the implant-tissue interface, protein adsorption from the biological environment and cell colonization [3,4,5,6]. Thus, topographic features at the protein/metal surface interface can significantly affect the cell morphology and activity [7].

Titanium has closer mechanical properties to the bone tissue than other metallic biomaterials, such as 316 L stainless steel and Co-Cr alloys; besides, its allotropic transformation from the alpha to beta phase, when its temperature exceeds the *T_TRANSUS_* (882 °C), is outstanding [8,9]. This transformation provides microstructural changes with a better mechanical performance compared to other metals. A second feature is that titanium can be alloyed with many elements modifying its *T_TRANSUS_*, and improving its mechanical strength, fatigue and wear resistance, among other properties. A wide range of titanium alloys could be manufactured depending on the proportion of alpha, beta and neutral alloying elements [10]. Another characteristic, and perhaps the most important, is that Ti becomes passivated, due to the formation of an oxide that protects the metal surface by isolating it from the corrosive medium, such as the biological environment, and enhancing its corrosion protection. Passivation is a phenomenon that is susceptible to the nature and surface changes of the substrate, which arise special interest in the development of new biomaterials. In several studies of Ti-CP and Ti6Al4V, Ti29Nb13Ta, Ti5Al2Nb1Ta alloys, which are the most frequently used as biomaterials, the chemical composition of the passive oxide depends on both the alloying elements and the corrosive medium [11,12,13,14,15,16,17,18]. This fact has an important impact in terms of corrosion resistance and biocompatibility and subsequently, it will affect osseointegration.

A comparison between titanium alloys, and some of their applications can be noted in the medical field. Even though Ti-CP has been used as biomaterial, it cannot be mechanically strengthened through heat treatments. Likewise, Ti-alpha alloys with a 30% of alpha stabilizer have no medical application, but they are used for aerospace applications because of their resistance to high temperatures. However, Ti-beta alloys, despite being biocompatible, show high density and low resistance to wear and fatigue, whereas the alpha-beta Ti alloys mainly constituted by 6% of alpha and 4% of beta stabilizer, have their main disadvantage in the toxicity of some alloying elements, such as V. In order to inhibit and/or decrease the effect of these elements, heat treatments had been suggested, leaving them farther away from the surface so as not to be in contact with organs and/or tissues; however, few studies have addressed the issue of how microstructural changes in these alloys immersed in biological environments may affect the cell adhesion, proliferation, morphology and osseointegration [19,20]. Specifically, Ti6Al4V can be subjected to heat treatments below and above its transformation temperature (*T_TRANSUS_* = 980 ± 20 °C) [8,9,21], and depending on the cooling rate it may provide different microstructures, such as globular, martensitic, bimodal and lamellar types. These microstructures can lead to modifications in the growth of passive oxide, its topography, chemical composition [22] and physicochemical surface changes, which could modify its electrochemical behavior at material/tissue interface [23,24], as well as cell morphology, adhesion, proliferation and differentiation [25].

Saos-2 osteoblast cells adhesion on Ti6Al4V alloys with different microstructures was studied in a previous work, where it was possible to observe that differences in the cellular morphology depended on TiO_2_ hydration and microstructure, whereas the ability of the cells to adsorb on the surface depended on the orientation of proteins and the chemical composition of extracellular matrix excreted by the cells. Cell coverage on these alloys was larger for Ti6Al4V as received than for Ti6Al4V_800_ and Ti6Al4V_1050_ alloys occurring in discrete alpha-phase (HCP) regions [26]. Recently different papers have focused on studying the adhesion, differentiation and proliferation of stem cells at nanoscale dimensions. Culture substrates with randomly ordered and smooth nanotopography induced elongation of human and rat Mesenchymal stem cells (MSCs). These results indicate that nanotopography plays a crucial role in osteogenic differentiation, and the biochemical cue of signal peptide is dependent on this. Therefore, the topographical cue is more important than proteins affecting the osteoblastic differentiation of induced stem cells. Hence, the ordered nanotopographical pattern changed the shape of resident cells by inducing cell elongation and eventually stimulated osteoblastic cell differentiation [27]. Other studies with biomimetic bone substitutes of collagen/silk (bi-template materials) showed the attachment and proliferation of bone marrow mesenchymal stem cells (BMSCs) in comparison with single template materials. The expression of relevant osteogenic genes of osteocalcin and osteonectin further confirmed that the BMSCs differentiate into osteoblast lineage. The bi-template materials combined with BMSCs present better biocompatibility and ability of new bone formation by in vivo assays [28,29,30]. In addition, during fracture healing, bone is formed by a cascade of events, such as gradual stiffening of the forming tissue and tissue deformation. Thus, mechanical loading of bone has been widely studied in bone tissue engineering by mechanical load stimulation on silk fibroin scaffolds, because it has an effect on mesenchymal stromal cells proliferation and differentiation, while the stress and shear stresses of the mechanical loading induce the osteogenic differentiation [31]. Therefore, it had been stated that cell adhesion, differentiation and proliferation differ depending on the substrate surface features, because these features modify the shape of the cells and their order and elongation on the different nano, meso and/or micro patterns, which has an effect on biocompatibility and subsequently, their osteointegration.

In this work, Ti6Al4V alloys subjected to 800 and 1050 °C with globular and lamellar microstructures were immersed in a culture medium for 7 days at 37 °C in the presence of L929 fibroblast cells, with the purpose of studying the effect of microstructure on cell adhesion and proliferation. These Ti alloys were characterized by SEM/EDX, while their passivation and corrosion resistance were characterized in situ by electrochemical techniques.

## 2. Results and Discussion

### 2.1. Microstructural Characterization

**Figure 1:**
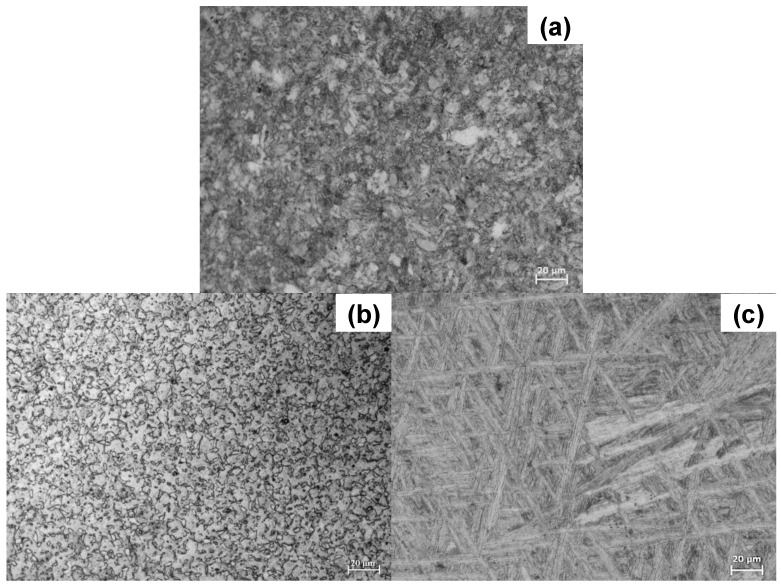
Optical micrographs of: (**a**) Ti6Al4V as received, (**b**) Ti6Al4V_800_ and (**c**) Ti6Al4V_1050_.

Figure 1 shows the microstructure of Ti6Al4V as received, as well as of Ti6Al4V_800_ and Ti6Al4V_1050_ obtained after the heat treatments. In the as received condition (Figure 1a), beta-phase equiaxed grains (dark zones) dispersed in the alpha-phase matrix (light zones) can be observed; meanwhile, in Ti6Al4V_800_ (Figure 1b), the alpha-phase acted as a barrier, thickening and preventing the grain size of the beta-phase (between 1 and 5 μm) from increasing rapidly [8,9,10]. On the other hand, in Ti6Al4V_1050_ alloy (Figure 1c) a Widmanstätten or lamellar-like microstructure can be seen, where both alpha- (light zones) and beta-phases (dark zones) form 1–2 μm thick and 40–80 μm long sheets, which are interspersed over the entire surface [32,33].

From XRD analysis, αTi and βTi phases were detected for Ti6Al4V as received and Ti6Al4V_800_ alloys, while for Ti6Al4V_1050_, the acicular alpha phase is generated. The β phase remains stable in the alloy after the heat treatment at 1050 °C and the alloying elements (Al and V) are distributed through the globular and lamellar microstructures depending on the heat treatment temperature and its cooling rate [26,34,35,36,37].

### 2.2. In Vitro Assays

All Ti6Al4V alloys were sterilized and analyzed by XPS, before the assays. The passive oxide was mainly composed of TiO_2_ and to a lesser extent, of suboxides, such as TiO and Ti_2_O_3_; these latter were in smaller proportion in Ti6Al4V_1050_ alloy. Furthermore, in heat-treated alloys, Ti6Al4V_800_ and Ti6Al4V_1050_, Al_2_O_3_ was observed being partially hydrated and showing the highest oxide thickness (4.8 and 5 nm, respectively), as compared to the untreated Ti6Al4V alloy (2 nm). V was not detected in any of the alloys [16,26,38,39,40,41,42,43]. These surfaces were seeded with 10,000 fibroblast cells to carry out in vitro cell proliferation assays for 7 days.

**Figure 2:**
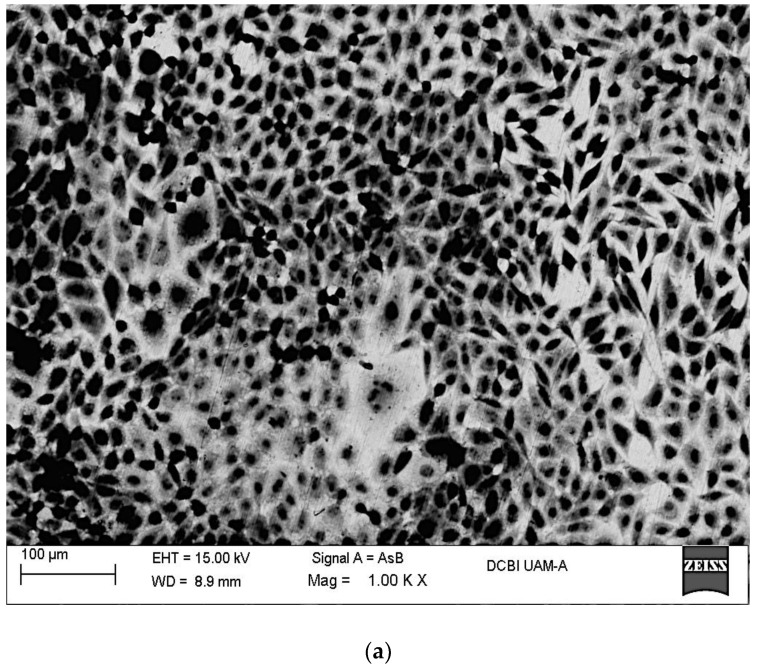

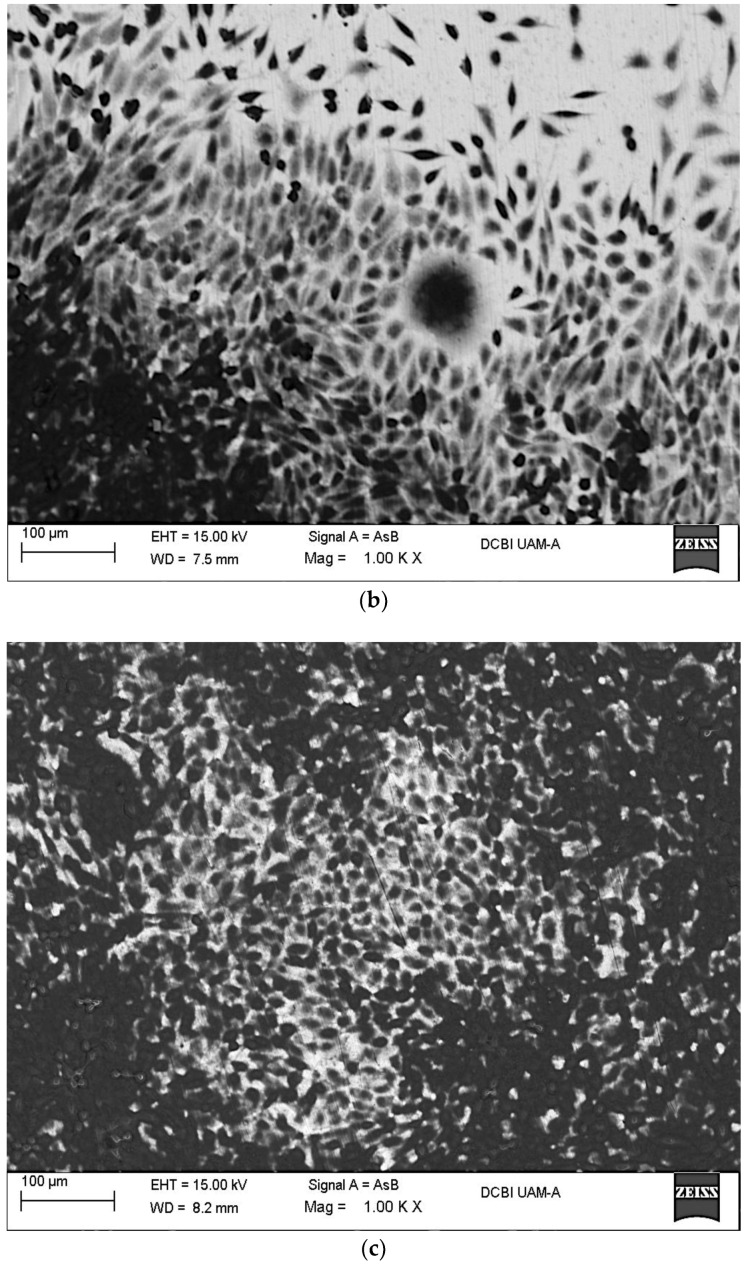
General view of the fibroblast cells adhered on: (**a**) Ti6Al4V as received alloy, (**b**) Ti6Al4V_800_ alloy and (**c**) Ti6Al4V_1050_ alloy after 7 days of immersion in DMEM 10% of FBS.

Figure 2a–c show SEM images recorded after the immersion of Ti6Al4V, Ti6Al4V_800_ and Ti6Al4V_1050_ alloys. The presence of fibroblast cells (dark spots) is evident on the different Ti6Al4V alloys showing a good biocompatibility. Adhered cells display polygonal shape, and are interconnected between each other regardless of the heat treatment. In certain zones, cells have accumulated forming small round islands whose dimensions range between 50 and 150 μm (Figure 2a,b), for Ti6Al4V and Ti6Al4V_800_ respectively. Meanwhile, in Ti6Al4V_1050_ alloy (Figure 2c), the cells are randomly dispersed over the entire surface forming more than one monolayer, which indicates a rapid proliferation. It is important to note that clustering cells are smaller on Ti6Al4V than those observed on Ti6Al4V_800_, likely related to the globular microstructure of both alloys and the presence of fine grains in the as received condition (Figure 1).

Cellular proliferation seems to be related to the way in which the passive oxide grows on different microstructures. Cell growth-like islands are observed on the alloys with globular microstructure where elements, such as Al, adsorbed water and/or OH^−^ are present. This behavior had also been observed in a previous work studying the osteoblast adhesion on Ti6Al4V_800_ alloy [26]. Contrary to the lamellar microstructure in Ti6Al4V_1050_, the alloying elements could be homogeneously distributed in the alpha and beta phases, avoiding the formation of these islands (Figure 2c), which results in a larger cell coverage throughout the surface as a consequence of a uniform and homogeneous passive oxide film growth over the entire surface (Ti6Al4V_1050_).

**Figure 3:**
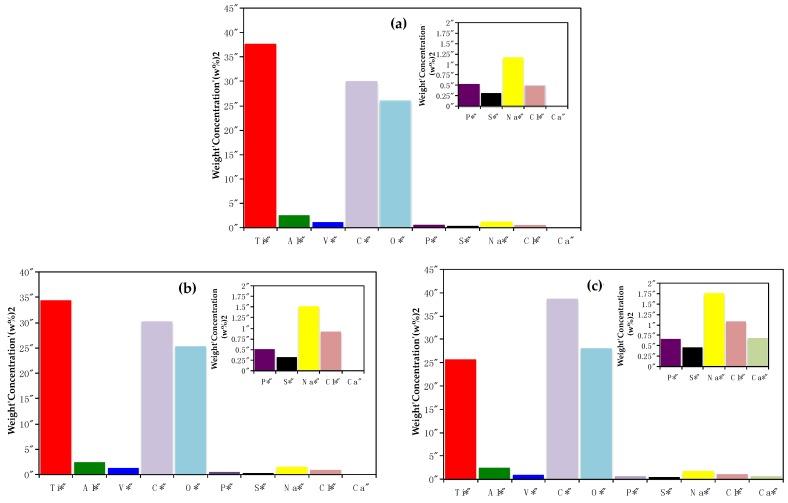
Energy Dispersive X-ray Spectroscopy (EDX) analysis of fibroblast cells adhered on: (**a**) Ti6Al4V as received alloy, (**b**) Ti6Al4V_800_ alloy and (**c**) Ti6Al4V_1050_ alloy after 7 days of immersion in DMEM 10% of FBS + fibroblast cells.

According to these images, a rapid proliferation of fibroblast cells towards the heat-treated alloys is observed, contrary to osteoblastic cells adhered on these Ti alloys [26]. Figure 3 shows the EDX analysis of fibroblast cells adhered on Ti6Al4V as received and heat-treated alloys after 7 days of immersion in cell culture medium. Different elements from the cells, their activity, and oxidation of the metal substrate were identified. Ti and O elements are mainly associated with the metallic oxide (TiO_2_), however much of the Ti signal comes from the alloy, because the percentage of this element in relation to Al and V is very high. The presence of Ti also indicates that the oxide thickness is small in the order of nanometers (see XPS technique). Instead, O signal comes not only from the passive oxide but also from the cells, as well as the C signal. These elements are part of the organic compounds and constitute the essential substance of the extracellular matrix, e.g., hyaluronic acid, glucosaminoglycans, collagen and elastin, which in their chemical structure involve OH^−^, –COOH, and –SO_3_^−^ groups. It is possible to consider a strong interaction between these chemical species and the point defects or vacancies in the TiO_2_ matrix [25,32]. The passive behavior of this oxide is due to the mobility of oxygen and hydroxide ions through defects (anion vacancies, $V_{\overset{¨}{O}}$ and $V_{\dot{O}H}$) which are positively charged [44,45,46,47,48,49,50], facilitating their adsorption. It is presumed that this adsorption process occurs in a similar way on different titanium alloys, because the percentages of these elements are in the same order. It is important to note the presence of P in the alloys, which could be incorporated and/or adsorbed into the passive oxide during immersion. To account for this fact, given the presence of hydroxyl groups on the surface that facilitates the anchoring of calcium ions and later phosphate ions, the likely precipitation of phosphates has been reported [51,52,53,54,55,56,57,58,59]. This assumption is consistent with the partial hydration of the passive film, related to the presence of alloying elements in the outermost surface, mainly Al [12,13,14,15,16,17,18,60,61,62,63]. This phenomenon seems to occur rapidly on Ti6Al4V_1050_ taking into account the larger cell coverage on the surface and the Ca signal recorded for this alloy. As mentioned above, the incorporation of Ca is mainly due to the TiO_2_ hydration evidenced in a previous work by XPS analysis, which studied osteoblast cells adhesion [26,64,65,66].

As a part of the extracellular matrix S, Na and Cl elements were identified on the three surfaces. The presence of S could be associated with the SO_3_^−^ groups that are provided by proteoglycans; these organic compounds are hydrophilic structures and attract cations, particularly Na^+^ (Figure 3b,c). The adsorption and orientation of these organic compounds on Ti6Al4V alloys’ surfaces depend on the passive oxide and its hydration [67,68,69]. Although cell adhesion is observed on the three alloys (Figure 2a–c), the confluency/proliferation is higher for Ti6Al4V_1050_, as well as C and O percentages, being minor the signals from the base metal (low Ti content for example, Figure 3c).

It had been established that this process begins with the adsorption of proteins from the culture medium on the passive oxide, which is probably defective and highly hydrated [60,70,71,72,73,74]. Additionally, different surface phenomena, such as pH and the interaction of different functional groups from the extracellular matrix with the passive oxide, also take place, as we mentioned earlier. It had been reported that at pH between 6.4 and 6.9, phosphate and OH^−^ ions are preferentially attracted to the outermost surface, where Ca is subsequently anchored to form calcium phosphate or a Ca-Ti-P compound [74,75,76]. Depending on the cells used in the biological assays, the components of the extracellular matrix participate in the precipitation processes of Ca and P. In this manner, in assays with osteoblast cells a low or no precipitation of Ca over the passive oxide grown on the Ti6Al4V as received had been reported. The same event occurs in the presence of fibroblast cells after 28 days of immersion [77]. This latter was also observed in a previous study after 7 days of immersion in cultures containing osteoblast cells [26], and in the present study. This indicates that the microstructure feature has a direct effect on the growth of the passive oxide, its hydration and interaction with cells and Ca precipitation [25,77,78].

Based on the above, cell adhesion occurs in different microstructures despite differences in hydration and passive oxide thickness. The fibroblast cells adsorption, as compared to osteoblast cells, is faster likely due to the strong interaction of the compounds derived from the extracellular matrix with the proteins adsorbed on the oxide. As was mentioned above, the interactions of OH^−^, –COOH, and −SO_3_^−^ groups through TiO_2_ enhance the diffusion of oxygen and hydroxyl ions modifying its resistive behavior; meanwhile, in the presence of osteoblast cells chemical compounds with large molecular weight (OH^−^, –COOH) form to a lesser extent and their adsorption is slow. Similar findings had been reported in the literature [16,25,79].

It can be summarized that in in vitro studies, no significant differences in cell morphology have been observed, although cell adhesion and proliferation have been higher in Ti6Al4V_1050_ alloy. This could be attributed to its microstructure (Figure 1), which entails a growth and chemical composition of the passive oxide film on the heat-treated metal substrate at 1050 °C. Besides, XPS analysis shows a minor content of TiO and Ti_2_O_3_ suboxides, an increase in the thickness and hydration, and the presence of Al_2_O_3_ for this Ti alloy [26]. It is important to point out that this work can be used to study the effect of the metallic microstructures on the stem cell fate.

### 2.3. Electrochemical Characterization

#### 2.3.1. Evolution of Open Circuit Potentials (E_OCP_)

**Figure 4:**
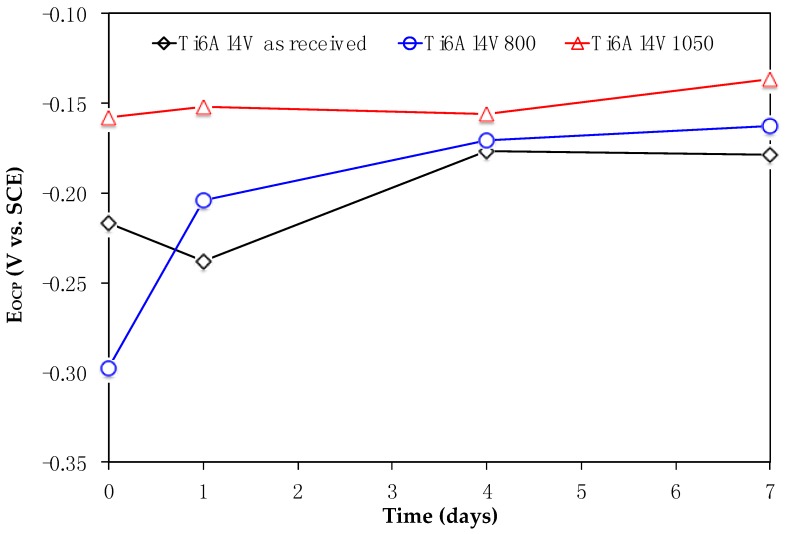
Open Circuit Potentials evolution (*E_OCP_*) for Ti6Al4V alloys immersed in DMEM 10% of FBS + fibroblast cells.

Figure 4 shows *E_OCP_* values recorded through the immersion of Ti6Al4V, Ti6Al4V_800_ and Ti6Al4V_1050_ in culture medium. At the initial time (day 0, no cells), the potentials are less negative in the following order: Ti6Al4V_800_, Ti6Al4V as received, Ti6Al4V_1050_. The difference in these potentials could be due to hydration of TiO_2_, thickness and likely protein adsorption [25,26]. In the presence of cells, the potential becomes less negative as the immersion increases, mainly for Ti6Al4V as received and Ti6Al4V_800_ alloys, meanwhile for Ti6Al4V_1050_, it remains almost constant varying from −0.160 to −0.145 V vs. SCE, thus indicating a steady state from the beginning of the test until the cell adhesion. Unlike this alloy, the potentials for Ti6Al4V as received and Ti6Al4V_800_ shift to positive values, reaching similar values for 4 and 7 days. These results could indicate that all Ti6Al4V alloys reach the same interfacial equilibrium of fibroblast cells adhered to the passive oxide immersed in the culture medium.

#### 2.3.2. Electrochemical Impedance Spectroscopy (EIS)

**Figure 5:**
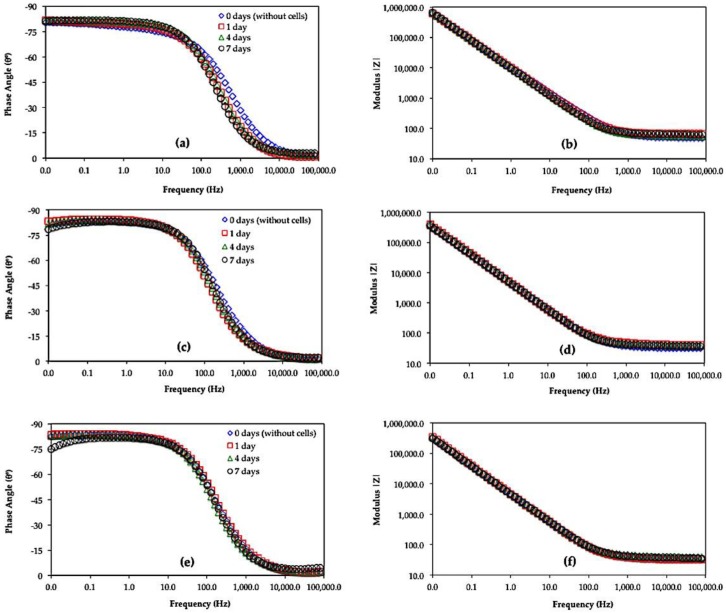
Bode diagrams (Phase angle vs. Frequency and Module |Z| vs. Frequency) obtained for the alloys: (**a**,**b**) Ti6Al4V as received, (**c**,**d**) Ti6Al4V_800_ and (**e**,**f**) Ti6Al4V_1050_ immersed in DMEM 10% of FBS + fibroblasts cells at 37 °C, pH 7.40 and 5% CO_2_.

**Figure 6:**
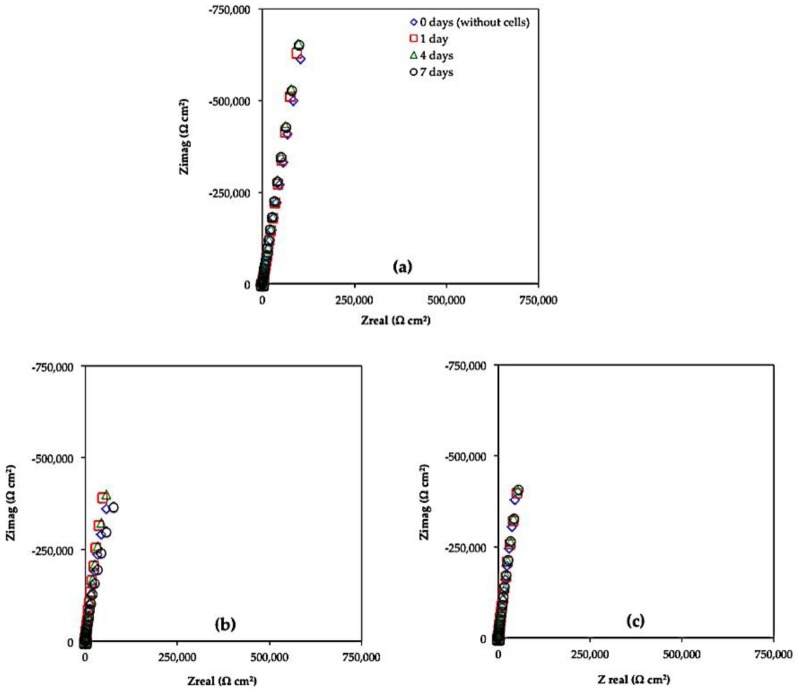
Nyquist diagrams (*Z_imag_* vs. *Z_real_*) obtained for alloys: (**a**) Ti6Al4V as received, (**b**) Ti6Al4V_800_ and (**c**) Ti6Al4V_1050_, immersed in DMEM 10% of FBS + fibroblast cells for 7 days at 37 °C, pH 7.40 and 5% CO_2_.

Figure 5 and Figure 6 show the Bode (Phase Angle vs. Frequency and Module |Z| vs. Frequency) and Nyquist (*Z_imag_* vs. *Z_real_*) diagrams for Ti6Al4V as received, Ti6Al4V_800_ and Ti6Al4V_1050_ immersed for 0, 1, 4 and 7 days in the culture medium with L929 fibroblasts cells. In these Figures, a similar electrochemical behavior is observed for the three alloys. In the phase angle diagrams (Figure 5), the maxima reach values close to −90, within 10^−2^ to 100 Hz interval; meanwhile, only slopes are recorded in the module plots at the same frequency interval, denoting the capacitive behavior of the alloys. In the Nyquist diagrams, the capacitive domain is related to the increase in *Z_imag_* for Ti6Al4V as received, Ti6Al4V_800_ and Ti6Al4V_1050_; whence the heat-treated alloys show minor impedance values likely due to the adhesion of the cells as the immersion progresses. The minor variations in EIS diagrams during the immersion can be related to the passive behavior of the oxide film mainly composed of TiO_2_ [11,12,13,14,15,16,17,18,42,43] and to a lesser extent, adsorption of water dipoles and/or proteins, as well as to adhesion of extracellular matrix as part of the metabolism of fibroblast cells until their adhesion [25,44]. However, these interfacial processes could explain whether the minor phase angle values recorded at low frequencies (Figure 5c,e) occur simultaneously, merging different time constants in EIS diagrams.

The deconvolution of these phenomena had been reported through Bode diagrams during in vitro assays of Ti6Al4V alloy in the presence of osteoblastic cells [25,44,80]. In particular, different slopes recorded in the module plots at high frequencies (10^5^ to 10^3^ Hz) suggest the contribution of the adsorbed cells/extracellular matrix interface, followed by the extracellular matrix/adsorbed proteins interface at intermediate frequencies, and the passive oxide film contribution at low frequencies [81,82,83]. However, in the present work EIS diagrams are poorly defined to discern these processes and are similar to those reported in the presence of osteoblast cells. The impedance values are, however, quite different.

Taking into account the minor variations in EIS diagrams and the fibroblast cell adhesion during the immersion (Figure 2a–c), protein adsorption, ion diffusion, including the precipitation of organic compounds at the oxide film-solution interface, cannot be discarded as part of the biomaterial osseointegration.

In this work, it is considered that the impedance values decrease as the mobility of different species (oxygen, hydroxide, calcium, and phosphorus, among others) through the oxide, is enhanced due to the interaction of the cells favoring its adhesion. These phenomena occurring at the interface facilitate the cellular deposition and growth and the biocompatibility on the three alloys, minimizing the influence of heat treatments. High cell adhesion is presumed for the heat-treated titanium alloys, since they are highly hydrated and less defective in comparison with Ti6Al4V as received. This fact is in agreement with SEM images shown in Figure 2; where a high cell adhesion is evident for Ti6Al4V_800_ and Ti6Al4V_1050_ alloys (Figure 2b,c).

**Figure 7:**
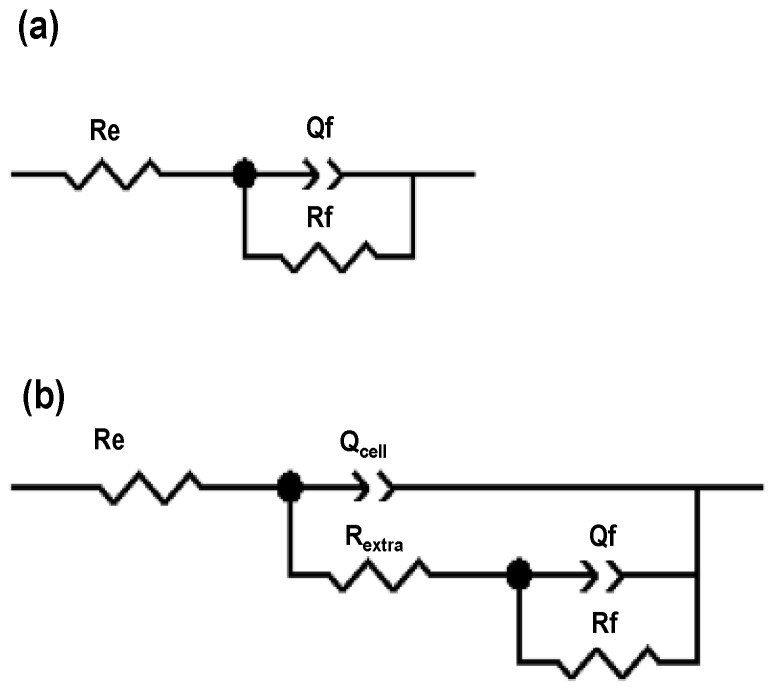
Equivalent circuits used to simulate experimental EIS responses of Ti6Al4V as received, Ti6Al4V_800_ and Ti6Al4V_1050_, immersed in DMEM %10 of FBS+ fibroblast cells for: (**a**) 0 days and (**b**) 1, 4 and 7 days.

To further study the electrochemical behavior of Ti alloys in the culture medium in the presence of fibroblast cells, the experimental impedance diagrams were simulated using the equivalent circuits shown in Figure 7. The RC equivalent circuit (Figure 7a) shows a constant phase element *Q_f_*, which simulates a non-linear behavior of the capacitor due to the passive oxide film-adsorbed proteins and the associated resistance, *R_f_*. A second RC arrangement, consisting of a constant phase element *Q_cell_* and *R_extra_*, was simulated for 1, 4 and 7 days, simulating the contribution of a non-linear behavior of the capacitor and the resistance of the film formed by the extracellular matrix and fibroblast cells, Figure 7b; this contribution is due to cell adhesion covering most part of the passive oxide [57,58,59].

Table 1 shows parameter values obtained from the adjustment of the experimental EIS diagrams of Ti6Al4V as received, Ti6Al4V_800_ and Ti6Al4V_1050_ alloys after the immersion. In the absence of cells (0 day), a minimal variation in the solution resistance (~47.68 Ω cm^2^) can be seen for the heat-treated alloys; meanwhile, *R_f_* and *Q_f_* values remained in the same order of magnitude (10^8^ Ω cm^2^ and 10^−5^ F cm^−2^). In the presence of cells (days 1, 4 and 7), similar *Re* values are observed for Ti6Al4V_800_ and Ti6Al4V_1050_; except for Ti6Al4V as received increasing from 10 to 18 Ω, which could be associated with minor changes in ion concentration in the culture medium, e.g., the precipitation of Na, Cl, S, Ca and P during cell adhesion process [26,32,60,61,62]. The *Q_f_* parameter decreases two orders of magnitude for *t* > 0, which could be due to modifications in the chemical composition of the oxide film and/or protein adsorption, showing a greater dielectric capacity of the surface; meanwhile, *R_f_* values are in the order of 10^8^ Ω cm^2^ for the different alloys during immersion, consistent with the formation of TiO_2_ in DMEM 10% of FBS + fibroblast cells [26,32].

The cell adhesion can be analyzed based on the results from the *R_extra_-Q_cell_* arrangement (see Table 1). *Q_cell_* values are in the same order of magnitude (10^−5^), which could be due to the adsorption of proteins and/or extracellular matrix excretion by the fibroblasts; this latter is composed mainly of collagen type I, proteoglycans linked to glucosaminoglycans, fibronectin, elastin and laminin allowing the fibroblasts adhesion [63]. The extracellular matrix resistances (*R_extra_*) on the TiO_2_ film/adsorbed proteins interface reach 10^3^ Ω cm^2^ for the three Ti alloys (day 1), whereas at 4 and 7 days, these values decrease. This fact could be related to the desorption of proteins and organic compounds from the culture medium and the formation of the extracellular matrix enhancing the cell adhesion, which leads to an increase in ionic mobility through TiO_2_ as was suggested in EIS diagrams [84,85]. Under this assumption, a larger coverage of the extracellular matrix and proliferation of fibroblast cells are predicted, in the following order: Ti6l4Vas received, Ti6l4V_800_ and Ti6l4V_1050_ as was shown in Figure 2.

## 3. Materials and Methods

### 3.1. Heat Treatments

Ti6Al4V alloy rods (Goodfellow Materials Ltd., Huntingdon, UK) of 12.7 mm in diameter and 20 mm in length were used; this alloy was encapsulated in quartz under an argon atmosphere in order to avoid its oxidation. 

Two heat treatments were performed for separate at two temperatures, one at 800 °C (named Ti6Al4V_800_) and the other one at 1050 °C (named Ti6Al4V_1050_), below and above Ti6Al4V transformation temperature (980 ± 20 °C), respectively [8]. These alloys remained inside the furnace for 6 h and were air-cooled at room temperature. Afterwards, discs of 2 mm thick were cut.

### 3.2. Metallography

In order to reveal the microstructure, a standard metallographic technique was used, consisting of its grinding with SiC paper sheet, polishing to a mirror finish and etching with Kroll reagent (HF + HNO_3_ and deionized water, 1:3:96) [9]. The microstructure was observed with a Nikon EPIPHOT 300 optical microscope coupled to a Nikon FDX-35 camera (Nikon Instruments Europe B.V., Amsterdam, The Netherlands).

### 3.3. X-ray Diffraction and XPS Characterizations

Phases on Ti6Al4V alloys, mechanically polished to a mirror finish, were identified by X-ray diffractometer, Brucker AXS model D8 Focus (Fison Instruments, East Grinstead, UK), with Kα radiation of Cu equipped with fluorescence filter of iron, in a range of 20 to 90°, at a velocity of 8° min^−1^, with a pitch of 0.02.

Prior to electrochemical measurements, Ti6Al4V alloys were mechanically polished using emery papers grade 1500 until obtaining homogeneous surfaces. In order to eliminate any contamination, the materials and electrochemical cell were sterilized in an autoclave for 30 min at 120 °C and 1.2 kg cm^−2^. After sterilization and prior to the in vitro assays, metal surfaces were analyzed with a Fisons MT500 Spectrometer, which was equipped with a hemiespheric electron analyzer (CLAM 2) and an X-ray source employing Mg Kα radiation (1253.6 eV) and operating at 300 W (Fison Instruments, East Grinstead, UK). The residual pressure in the analysis chamber was kept below 10^−8^ Torr during the measurements. Spectra were recorded using a 20 eV pass-through energy, which is typical for high-resolution conditions. A background subtraction was carried out using the Shirley method, fixing the experimental curve to a mixture of Gaussian and Lorentzian curves of variable proportion. The binding energy of C1s from the contamination of saturated hydrocarbons to 285.0 eV was used as internal reference to calibrate each spectrum.

### 3.4. In Vitro Assays

DMEM 10% of FBS was used as a culture medium, which was prepared as follows: 1 mL 200 mM L-Glutamine Gibco^®^ (Darmstadt, Germany), 1 mL Gibco^®^ Penicillin-Streptomycin, 1 mL Gibco^®^ Sodium Pyruvate and 10 mL FSB (Fetal Bovine Serum, Sigma-Aldrich^®^, St. Louis, MO, USA) were added to 90 mL DMEM (Dulbecco’s Modified Medium, Gibco^®^) under stirring conditions. Ti6Al4V as received, Ti6Al4V_800_ and Ti6Al4V_1050_ discs were immersed in the culture medium for 24 h (day 0, in the absence of cells). Afterwards, 10,000 L929 fibroblast cells provided by the Biological Research Center, Madrid, Spain, were seeded on each surface using a pippete.

The electrochemical measurements were performed using a homemade electrochemical cell [27], with Ti6Al4V as received, Ti6Al4V_800_ and Ti6Al4V_1050_ discs (area 1.25 cm^2^) as working electrodes, a saturated calomel electrode as reference and a platinum wire (Goodfellow Cambridge Ltd., Huntingdon, UK) as a counter electrode. Luer unions were fitted to control the entry and exit of 5% CO_2_.

Open circuit potentials (*E_OCP_*) were measured for 600 s, and immediately the Electrochemical Impedance Spectroscopy (EIS) characterization was carried out, applying a sinusoidal signal 5 mV of amplitude in a frequency range of 10^5^ to 10^−2^ Hz and 10 points per decade, using a Gamry series 600 Potentiostat-Galvanostat coupled to a PC for the control and data acquisition. All assays were performed in triplicate. *E_OCP_* and EIS measurements were performed at 0, 1, 4 and 7 days and the materials were maintained in the culture medium + cells for 7 days at 37 °C, pH = 7.40, 5% CO_2_ and 100% relative humidity. The culture medium was renewed every 48 h to supply nutrients and eliminate cell waste products.

### 3.5. Cell Fixation

After in vitro assays, fibroblast cells were fixed onto the metal surfaces by adding 1 mL of 2% glutaraldehyde and maintained at 4 °C for 24 h. Subsequently, a dehydration process was carried out by immersion in a sequence of solutions ranging from 35% to 100% of ethanol. Thereafter, a 50% Trimethylsilane (TMS Sigma-Aldrich^®^) solution (0.5 mL TMS in 0.5 mL 100% ethanol) was added to the cells for 10 min. This solution was withdrawn and 1 mL of 100% TMS was added over 10 min. Finally, the TMS was removed and allowed to air dry for 30 min.

### 3.6. SEM-EDX Surface Characterization

After cell fixation, the surfaces were characterized by SEM/EDX using a Zeiss SUPRA 55-VP Scanning Electron Microscope (Carl Zeiss de México S.A. de C.V., Mexico, Mexico), operating at 15 kV and coupled to an EDX for microanalysis capability.

## 4. Conclusions

Fibroblast cells show biocompatibility on Ti6Al4V as received and alloys heat-treated at 800 °C and 1050 °C, namely Ti6Al4V_800_ and Ti6Al4V_1050_, respectively, after seven days of immersion in DMEM 10% of FBS. The differences in cell adhesion and morphology are characterized by SEM, where agglomerated cells are evident on Ti6Al4V as received and Ti6Al4V_800_, presumably due to the globular microstructure of these alloys; this contrasts with the lamellar microstructure of Ti6Al4V_1050_ that displays the largest cell coverage. As part of cell proliferation, the signals of C, O, P, S, Na and Cl, which constitute the essential substance of the extracellular matrix (e.g., hyaluronic acid, glucosaminoglycans, collagen and elastin) are detected by EDX analysis. In addition, the precipitation of Ca is only observed for Ti6Al4V_1050_ alloy. Finally, a similar electrochemical behavior is seen for Ti6Al4V alloys during immersion in the culture medium, related to the formation of TiO_2_, overlaying the contribution of protein adsorption and the precipitation of the extracellular matrix from the cells.

## Figures and Tables

**Table 1:** Parameters obtained from the experimental EIS (Electrochemical Impedance Spectroscopy) diagrams of Ti6Al4V as received, Ti6Al4V_800_ and Ti6Al4V_1050_ during 7 days of immersion in DMEM 10% of FBS+ fibroblast cells.

---	Time (Days)	*R_e_* (Ω cm^2^)	*R_extra_* (Ω cm^2^)	*Q_cell_* (Siemens s^n^) (cm^−2^)	*n*	*R_f_* (Ω cm^2^)	*Q_f_* (Siemens s^n^) (cm^−2^)	*N*	χ^2^
**Ti6Al4V**	0	49.82	---	---	---	1.07 × 10^8^	1.84 × 10^−5^	0.859	4.36 × 10^−3^
	1	67.29	6661	1.79 × 10^−5^	0.887	1.29 × 10^8^	8.47 × 10^−7^	0.978	4.40 × 10^−4^
	4	59.11	1995	1.79 × 10^−5^	0.900	1.34 × 10^8^	8.56 × 10^−7^	0.977	8.87 × 10^−4^
	7	66.33	1177	1.82 × 10^−5^	0.903	1.61 × 10^8^	8.62 × 10^−7^	0.993	4.84 × 10^−4^
**Ti6Al4V_800_**	0	34.33	---	---	---	1.17 × 10^8^	3.59 × 10^−5^	0.915	6.70 × 10^−3^
	1	45.14	8515	3.39 × 10^−5^	0.929	1.34 × 10^8^	4.47 × 10^−7^	0.991	6.7 0 × 10^−3^
	4	42.14	2866	3.28 × 10^−5^	0.926	1.64 × 10^8^	6.09 × 10^−7^	0.956	7.69 × 10^−3^
	7	39.40	1686	3.45 × 10^−5^	0.921	8.40 × 10^6^	1.92 × 10^−7^	0.937	5.75 × 10^−3^
**Ti6Al4V_1050_**	0	58.88	---	---	---	1.74 × 10^8^	3.46 × 10^−5^	0.927	4.77 × 10^−3^
	1	51.03	7760	3.28 × 10^−5^	0.923	1.06 × 10^8^	1.41 × 10^−7^	0.940	6.67 × 10^−3^
	4	44.89	1277	3.22 × 10^−5^	0.922	1.76 × 10^8^	1.59 × 10^−7^	0.939	5.09 × 10^−3^
	7	55.36	945	3.20 × 10^−5^	0.924	1.96 × 10^8^	1.87 × 10^−7^	0.942	9.35 × 10^−3^

## References

[1] Le Guéhennec L., Soueidan A., Layrolle P., Amouriq Y. (2007). Surface treatments of titanium dental implants for rapid osseointegration. Dent. Mater..

[2] Ratner B.D., Hoffman A.S., Schoen F.J., Lemons J.E. (2013). Biomaterials Science: An Introduction to Materials in Medicine.

[3] Scholz M.S., Blanchfield J.P., Bloom L.D., Coburn B.H., Elkington M., Fuller J.D., Gilbert M.E., Muflahi S.A., Pernice M.F., Rae S.I. (2011). The use of composite materials in modern orthopedic medicine and prosthetic device. Compos. Sci. Technol..

[4] Waite D.E. (1989). Overview and historical perspective of oral reconstructive surgery. Oral Surg. Oral Med. Oral Pathol..

[5] Lizuca T., Hallermann W., Seto I., Smolka W. (2006). A titanium arch bar for maxillomandibular fixation in oral and maxillofacial surgery. J. Oral Maxillofac. Surg..

[6] Castner D.G., Ratner B.D. (2002). Biomedical surface science: Foundations to frontiers. Surf. Sci..

[7] Sumita M. (1997). Present status and future trend of metallic materials used in orthopedics. Orthop. Surg..

[8] Vydehi Arun J. (2017). Titanium Alloys: An Atlas of Structures and Fracture Features, Physical Metallurgy of Titanium Alloys.

[9] Donachie M.J. (2000). Titanium a Technical Guide. Understanding the Metallurgy of Titanium.

[10] Vrancken B., Thijs L., Kruth J.P., van Humbeeck J. (2012). Heat treatment of Ti6Al4V produced by Selective Laser Melting: Microstructure and mechanical properties. J. Alloys Compd..

[11] Hanawa T., Asami K., Asaoka K. (1997). Repassivation of titanium and surface oxide film regenerated in simulated bioliquid. J. Biomed. Mater. Res..

[12] Li S.J., Yang R., Niinomi M., Hao Y.L., Cui Y.Y. (2004). Formation and growth of calcium phosphate on the surface of oxided Ti-29Nb-13Ta-4.6Zr alloy. Biomaterials.

[13] Rafieerad A.R., Ashra M.R., Mahmoodian R., Bushroa A.R. (2015). Surface characterization and corrosion behavior of calcium phosphate-base composite layer on titanium and its alloys via plasma electrolytic oxidation: A review paper. Mater. Sci. Eng. C.

[14] Niinomi M. (2003). Fatigue performance and cytotoxicity of low rigidity titanium alloy Ti-29Nb-13Ta-4.6Zr. Biomaterials.

[15] Tanaka Y., Nakai M., Akahori T., Niinomi M., Tsutsumi Y., Doi H., Hanawa T. (2008). Characterization of air-formed surface oxide film on Ti-29Nb-13Ta-4.6Zr alloy surface using XPS and AES. Corros. Sci..

[16] Variola F., Yi J.H., Richert L., Wuest J.D., Rosei F., Nanci A. (2008). Tailoring the surface properties of Ti6Al4V by controlled chemical oxidation. Biomaterials.

[17] De Assis S.L., Wolynec S., Costa I. (2006). Corrosion characterization of titanium alloys by electrochemical techniques. Electrochim. Acta.

[18] Lausmaa J., Kasemo B. (1990). Surface Spectroscopic Characterization on Titanium Implant Materials. Appl. Surf. Sci..

[19] Chen Q., Thouas G.A. (2015). Metallic implant biomaterials. Mater. Sci. Eng..

[20] Hee A.C., Zhao Y., Jamali S.S., Martin P.J., Bendavid A., Peng H., Cheng X. (2017). Corrosion behaviour and microstructure of tantalum film on Ti6Al4V substrate by filtered cathodic vacuum arc deposition. Thin Solid Films..

[21] Liu X., Chu P.K., Ding C. (2004). Surface modification of titanium, titanium alloys and related materials for biomedical applications. Mater. Sci. Eng..

[22] Williams D.F. (1981). Titanium and Titanium Alloys, Biocompatibility of Clinical Implant Materials.

[23] Goldberg J.R., Gilbert J.L. (2004). The electrochemical and mechanical behavior of passivated and TiN/AlN coated CoCrMo and Ti6Al4V alloys. Biomaterials.

[24] Ramires J., Guastaldi A.C. (2002). Study of Ti-6Al-4V biomaterial using electrochemistry and XPS techniques. Quim. Nova.

[25] Anselme K. (2000). Osteoblast adhesion on biomaterials. Biomaterials.

[26] Chávez-Díaz M.P., Escudero-Rincón M.L., Arce-Estrada E.M., Cabrera-Sierra R. (2017). Osteoblast Cell Response on the Ti6Al4V Alloy Heat-Treated. Materials.

[27] Wang J., Wang L., Yang M., Zhu Y., Tomsia A., Mao C. (2014). Untangling the Effects of Peptide Sequences and Nanotopographies in a Biomimetic Niche for Directed Differentiation of iPSCs by Assemblies of Genetically Engineered Viral Nanofibers. Nano Lett..

[28] Cao B., Yang M., Mao C. (2016). Phage as a Genetically Modifiable Supramacromolecule in Chemistry, Materials and Medicine. Acc. Chem. Res..

[29] Wang J., Yang Q., Mao C., Zhang S. (2012). Osteogenic differentiation of bone marrow mesenchymal stem cells on the collagen/silk fibroin bitemplate induced biomimetic bone substitutes. J. Biomed. Mater. Res. Part A.

[30] Vetsch J.R., Betts D.C., Müller R., Hofmann S. (2017). Flow velocity-driven differentiation of human mesenchymal stromal cells in silk fibroin scaffolds: A combined experimental and computational approach. PLoS ONE.

[31] Wang Q., Cao I., Liu Y., Zheng A., Wu J., Jiang X., Ji P. (2017). Evaluation of synergistic osteogenesis between icariin and BMP2 through a micro/meso hierarchical porous delivery system. Int. J. Nanomed..

[32] García-Alonso M.C., Saldaña L., Alonso C., Barranco V., Muñoz-Morris M.A., Escudero M.L. (2009). In situ cell culture monitoring on a Ti-6Al-4V surface by electrochemical techniques. Acta Biomater..

[33] Sallica-Leva E., Jardini A.L., Fogagnolo J.B. (2013). Microstructure and mechanical behavior of porous Ti-6Al-4V parts obtained by selective laser melting. J. Mech. Behav. Biomed. Mater..

[34] Elmer J.W., Palmer T.A., Babu S.S., Specht E.D. (2005). In situ observations of lattice expansion and transformation rates of and phases in Ti-6Al-4V. Mater. Sci. Eng. A.

[35] Malinov S., Guo Z., Sha W., Wilson A. (2001). Differential Scanning Calorimetry Study and Computer Modelling of β-α Phase Transformation in Ti-6Al-4VAlloy. Metall. Mater. Trans. A.

[36] Malinov S., Sha W., Guo Z., Tang C.C., Long A.E. (2002). Synchrotron X-ray diffraction study of the phase transformations in titanium alloys. Mater. Charact..

[37] Armstrong N.R., Quinn R.D. (1977). Auger and X-ray photoelectron spectroscopic and electrochemical characterization of titanium thin film electrodes. Surf. Sci..

[38] Strohmeier B.R. (1990). An ESCA method for determining the oxide thickness on aluminium-alloys. Surf. Interface Anal..

[39] Milosev M., Metikos-Hukovic M., Strehblow H.H. (2000). Passive film on orthopaedic TiAlV alloy formed in physiological solution investigated by X-ray photoelectron spectroscopy. Biomaterials.

[40] McCafferty E., Wightman J.P. (1999). An X-ray photoelectron spectroscopy sputter profile study of the native air-formed oxide film on titanium. Appl. Surf. Sci..

[41] Feliu S., Barranco V. (2003). XPS study of the surface chemistry of conventional hot-dip galvanised pure Zn, galvanneal and Zn–Al alloy coatings on steel. Acta Mater..

[42] Bunker B.C., Nelson G.C., Zavadil K.R., Barbour J.C., Wall F.D., Sullivan J.P. (2002). Hydration of Passive Oxide Films on Aluminum. J. Phys. Chem. B.

[43] Popa M.V., Demetrescu I., Vasilescu E., Drob P., Santana López A., Mirza-Rosca J., Vasilescu C., Ionita D. (2004). Corrosion susceptibility of implant materials. Ti-5Al-4V and Ti-6Al-4Fe in artificial extra-cellular fluids. Electrochim. Acta.

[44] Chikarakara E., Fitzpatrick P., Moore E., Levingstone T., Grehan L., Higginbotham C., Vázquez M., Bagga K., Naher S., Brabazon D. (2016). In vitro fibroblast and pre-osteoblastic cellular responses on laser surface modified Ti-6Al-4V. Appl. Surf. Sci..

[45] Chao C.Y., Lin L.F., Macdonald D.D. (1981). A Point Defect model for anodic passive films, I. Film growth kinetics. J. Electrochem. Soc..

[46] Cabrera-Sierra R., Hallen J.M., Vázquez-Arenas J., Vázquez G., González I. (2010). EIS characterization of tantalum and niobium oxide films based on a modification of the point defect model. J. Electroanal. Chem..

[47] Cabrera-Sierra R., Vázquez-Arenas J., Cardoso S., Luna-Sánchez R.M., Trejo M.A., Marín-Cruz J., Hallen J.M. (2011). Analysis of the formation of Ta_2_O_5_ passive films in acid media through mechanistic modeling. Electrochim. Acta.

[48] Göpel W., Anderson J.A., Frenkel D., Jeaning M., Philips K., Schäfer J.A., Rocker G. (1984). Surface defects of TiO_2_ (1 1 0): A combined XPS, XAES and ELS study. Surf. Sci..

[49] Castellani C., Lindtner R.A., Hausbrandt P., Tschegg E., Stanzl-Tschegg S.E., Zanoni G., Beck S., Weinberg A.M. (2011). Bone-implant interface strength and osseointegration: Biodegradable magnesium alloy versus standard titanium control. Acta Biomater..

[50] Contu F., Elsener B., Bohni H. (2002). Characterization of implant materials in fetal bovine serum and sodium-sulfate by electrochemical impedance spectroscopy-I-Mechanically polished samples. J. Biomed. Mater. Res..

[51] Chang E., Lee T.M. (2002). Effect of surface chemistry and characteristics of Ti6Al4V on the Ca and P adsorption and ion dissolution in Hanks ethylene diamine tetraacetic acid solution. Biomaterials.

[52] Roessler S., Zimmermann R., Scharnweber D., Werner C., Worch H. (2002). Characterization of oxide layers on Ti6Al4V and titanium by streaming potential and streaming current measurements. Colloids Surf. B.

[53] Einsenbarth E., Velten D., Müller M., Thull R., Breme J. (2004). Biocompatibility of beta-stabilizing of titanium alloys. Biomaterials.

[54] Frauchiger L., Taborelli M., Aronsson B.O., Descouts P. (1999). Ion adsorption on titanium surface exposed to a physiological solution. Appl. Surf. Sci..

[55] Khan M.A., Williams R.L., Williams D.F. (1999). The corrosion behavior of Ti-6Al-4V, Ti-6Al-7Nb and Ti-13Nb-13Zr in protein solutions. Biomaterials.

[56] Barranco V., Escudero M.L., García-Alonso M.C. (2011). Influence of the microstructure and topography on the barrier properties of oxide scales generated on blasted Ti6Al4V surfaces. Acta Biomaterialia..

[57] Feng K.-C., Wu E.-Y., Pan Y.-N., Ou K.-L. (2007). Effects of Chemical and Heat Treatments on Surface Characteristics and Biocompatibility of Titanium-Niobium Alloys. Mater. Trans..

[58] Lavos-Valereto I.C., Wolynec S., Ramires I., Guastaldi A.C., Costa I. (2004). Electrochemical impedance spectroscopy characterization of passive film formed on implant Ti6Al7Nb alloy in Hank’s solution. J. Mater. Sci..

[59] Kubies D., Himmlová L., Riedel T., Chánová E., Balík K., Doudeˇrová M., Bártová J., Pešáková V. (2011). The Interaction of Osteoblasts with Bone-Implant Materials: 1. The effects of physicochemical surface properties of implant materials. Physiol. Res..

[60] Chesmel K.D., Clark C.C., Brighton C.T., Black J. (1995). Cellular responses to chemical and morphologic aspects of biomaterial surfaces. II. The biosynthetic and migratory response of bone cell populations. J. Biomed. Mater. Res..

[61] Oates C.J., Wen W., Hamilton D.W. (2011). Role of Titanium Surface Topography and Surface Wettability on Focal Adhesion Kinase Mediated Signaling in Fibroblasts. Materials.

[62] Szymonowicz M., Korczynski M., Dobrzynski M., Zawisza K., Mikulewicz M., Karuga-Kuzniewska E., Zywicka B., Rybak Z., Wiglusz R.J. (2017). Cytotoxicity Evaluation of High-Temperature Annealed Nanohydroxyapatite in Contact with Fibroblast Cells. Materials.

[63] Alberts B., Johnson A., Lewis J., Raff M., Roberts K., Walter P. (2002). Molecular Biology of the Cell.

[64] Cabrera-Sierra R., Pech-Canul M.A., González I. (2006). The Role of Hydroxide in the Electrochemical Impedance Response of Passive Films in Corrosion Environments. J. Electrochem. Soc..

[65] Takana Y., Kobayashi E., Hiromoto K., Asami H., Imai H., Hanawa T. (2007). Calcium phosphate formation on titanium by low-voltage electrolytic treatments. J. Mater. Sci..

[66] Ban S., Maruno S. (1993). Deposition of calcium phosphate on titanium by electrochemical process in simulated body fluid. Jpn. J. Appl. Phys..

[67] Cremasco A., Dutra Messias A., Rodrigues Esposito E. (2011). Aparecida de Rezende Duek, R. Caram. Effects of alloying elements on the cytotoxic response of titanium alloys. Mater. Sci. Eng. C.

[68] Bargeron C.B., Givens R.B. (1980). Precursive blistering in the localized corrosion of aluminum. Corrosion.

[69] Bargeron C.B., Givens R.B. (1977). Localized Corrosion of Aluminum: Blister Formation as a Precursor of Pitting. J. Electrochem. Soc..

[70] Puleo D.A., Nanci A. (1999). Understanding and controlling the bone–implant interface. Biomaterials.

[71] Thompson G.J., Puleo D.A. (1996). Ti-6Al-4V ion solution inhibition of osteogenic cell phenotype as a function of differentiation time course in vitro. Biomaterials.

[72] Nichols K.G., Puleo D.A. (1997). Effect of metal ions on the formation and function of osteoclastic cells in vitro. J. Biomed. Mater. Res..

[73] Neupane M.P., Kim Y.K., Park S.I., Lee S.J., Lee M.H., Bae T.S. (2008). Effect of pH on the Structure and in vitro Osteoblasts Response to Anodic Titanium Oxide. Met. Mater. Int..

[74] Geetha M., Kamachi Mudali U., Gogia A.K., Asokamani R., Raj B. (2004). Influence of microstructure and alloying elements on corrosion behavior of Ti-13Nb-13Zr alloy. Corros. Sci..

[75] Lee B.H., Lee C., Kim D.G., Choi K., Lee K.H., Kim Y.D. (2008). Effect of surface structure on biomechanical properties and osseointegration. Mater. Sci. Eng. C.

[76] Geetha M., Durgalaksshmi D., Asokamani R. (2010). Biomedical Implants: Corrosion and its Prevention-A Review. Recent Pat. Corros. Sci..

[77] Mustafa K., Pan J., Wroblewsli J., Leygraf C., Arvidson K. (2002). Electrochemical impedance spectroscopy and X-ray photoelectron spectroscopy analysis of titanium surfaces cultured with osteoblast-like cells derived from human mandibular bone. J. Biomed. Mater. Res..

[78] Mróz W., Bunder B., Syroka R., Niedzielski K., Golanski G., Slósarczyk A., Schwarz D., Douglas T.E. (2014). In vivo implantation of porous titanium alloy implants coated with magnesium-doped octacalcium phosphate and hydroxyapatite thin films using pulsed laser deposition. J. Biomed. Mater. Res. Part B.

[79] Anselme K., Linez P., Bigerelle M., Le Maguer D., Le Maguer A., Hardouin P., Hildebrand H.F., Iost A., Leroy J.M. (2000). The relative influence of the topography and chemistry of TiAl6V4 surfaces on osteoblastic cell behaviour. Biomaterials.

[80] Karimzadeh F., Heidarbeigy M., Saatchi A. (2008). Effect of heat treatment on corrosion behavior of Ti-6Al-4V alloy weldments. J. Mater. Process. Technol..

[81] Wang X.J., Li Y.C., Lin J.G., Hodgson P.D., Wen C. (2008). Effect of heat-treatment atmosphere on the bond strength of apatite layer on Ti substrate. Dent. Mater..

[82] Farhang P., Pupak A., Sirous A. (2012). Influence of mechanical and chemical surface treatments on the formation of bone-like structure in cpTi for endosseous dental implants. Appl. Surf. Sci..

[83] He X., Zhang G., Wang X., Hang R., Huang X., Qin L., Tang B., Zhang X. (2017). Biocompatibility, corrosion resistance and antibacterial activity of TiO_2_/CuO coating on titanium. Ceram. Int..

[84] Burgos-Asperilla L., Garcia Fierro J.L., Gamero M., Escudero M.L., Alonso C., García-Alonso M.C. (2015). In situ electrochemical study of the interaction of cells with thermally treated titanium. Biointerphases.

[85] Burgos-Asperilla L., García-Alonso M.C., Escudero M.L., Alonso C. (2015). Cell adhesion on Ti surface with controlled roughness. Rev. Metal..

